# Mountain Pine Beetles Use Volatile Cues to Locate Host Limber Pine and Avoid Non-Host Great Basin Bristlecone Pine

**DOI:** 10.1371/journal.pone.0135752

**Published:** 2015-09-02

**Authors:** Curtis A. Gray, Justin B. Runyon, Michael J. Jenkins, Andrew D. Giunta

**Affiliations:** 1 Department of Wildland Resources, Utah State University, 5230 Old Main Hill, Logan, UT, 84322, United States of America; 2 USDA Forest Service, Rocky Mountain Research Station, Forestry Sciences Laboratory, Bozeman, Montana, United States of America; University of Nevada Reno, UNITED STATES

## Abstract

The tree-killing mountain pine beetle (*Dendroctonus ponderosae* Hopkins) is an important disturbance agent of western North American forests and recent outbreaks have affected tens of millions of hectares of trees. Most western North American pines (*Pinus* spp.) are hosts and are successfully attacked by mountain pine beetles whereas a handful of pine species are not suitable hosts and are rarely attacked. How pioneering females locate host trees is not well understood, with prevailing theory involving random landings and/or visual cues. Here we show that female mountain pine beetles orient toward volatile organic compounds (VOCs) from host limber pine (*Pinus flexilis* James) and away from VOCs of non-host Great Basin bristlecone pine (*Pinus longaeva* Bailey) in a Y-tube olfactometer. When presented with VOCs of both trees, females overwhelmingly choose limber pine over Great Basin bristlecone pine. Analysis of VOCs collected from co-occurring limber and Great Basin bristlecone pine trees revealed only a few quantitative differences. Noticeable differences included the monoterpenes 3-carene and D-limonene which were produced in greater amounts by host limber pine. We found no evidence that 3-carene is important for beetles when selecting trees, it was not attractive alone and its addition to Great Basin bristlecone pine VOCs did not alter female selection. However, addition of D-limonene to Great Basin bristlecone pine VOCs disrupted the ability of beetles to distinguish between tree species. When presented alone, D-limonene did not affect behavior, suggesting that the response is mediated by multiple compounds. A better understanding of host selection by mountain pine beetles could improve strategies for managing this important forest insect. Moreover, elucidating how Great Basin bristlecone pine escapes attack by mountain pine beetles could provide insight into mechanisms underlying the incredible longevity of this tree species.

## Introduction

The mountain pine beetle (MPB; Coleoptera: Curculionidae; *Dendroctonus ponderosae* Hopkins) is one of the most ecologically and socioeconomically important forest insects in North America. Outbreaks of this native insect during the early 21st century have been extensive, with over 3.5 million hectares of tree mortality in 2009 alone [1]. Such outbreaks can have important consequences for wildlife [2], forest carbon dynamics [3], nutrient cycling [4], wildfires [5], and have contributed to the rapid decline of some high elevation tree species [6,7].

MPBs kill trees by attacking *en masse* using a complex system of volatile semiochemicals involving multiple beetle-produced aggregation and anti-aggregation pheromones and host-produced kairomones [8]. Once in contact with a suitable host, pioneering females initiate mass attacks by oxidizing the host-produced monoterpene α-pinene to produce the aggregation pheromone verbenol [8]. Males arrive and produce *exo*-brevicomin which attracts more beetles. Host-produced monoterpenes including α-pinene [9], myrcene, and terpinolene [10] synergize the aggregation pheromones. In the latter stages of a mass attack, increased production of the anti-aggregation pheromone verbenone (via oxidation of verbenol) terminates host colonization [11]. Despite having a good understanding of the sophisticated chemical ecology underlying mass attacks, less is known about the cues used by pioneering females to locate trees [12]. The prevailing theory is that during the pre-aggregation phase females locate host trees using visual cues or through random landings [8,12]. Studies have reported MPB attraction to dark silhouettes and large, tree-shaped cylinders [13,14] suggesting a role for visual cues. Other studies have indicated that pioneering females intercept hosts at random which explains MPB’s preference for large diameter trees due to their larger surface area [15,16]. Conversely, there is evidence for the use of long distance sensing using volatile organic compounds (VOCs) by MPBs [17–19]. Plant VOCs emitted by trees are known to be used in host location by other bark beetle species [20], suggesting they might be similarly used in host location by MPBs.

**Fig 1:**
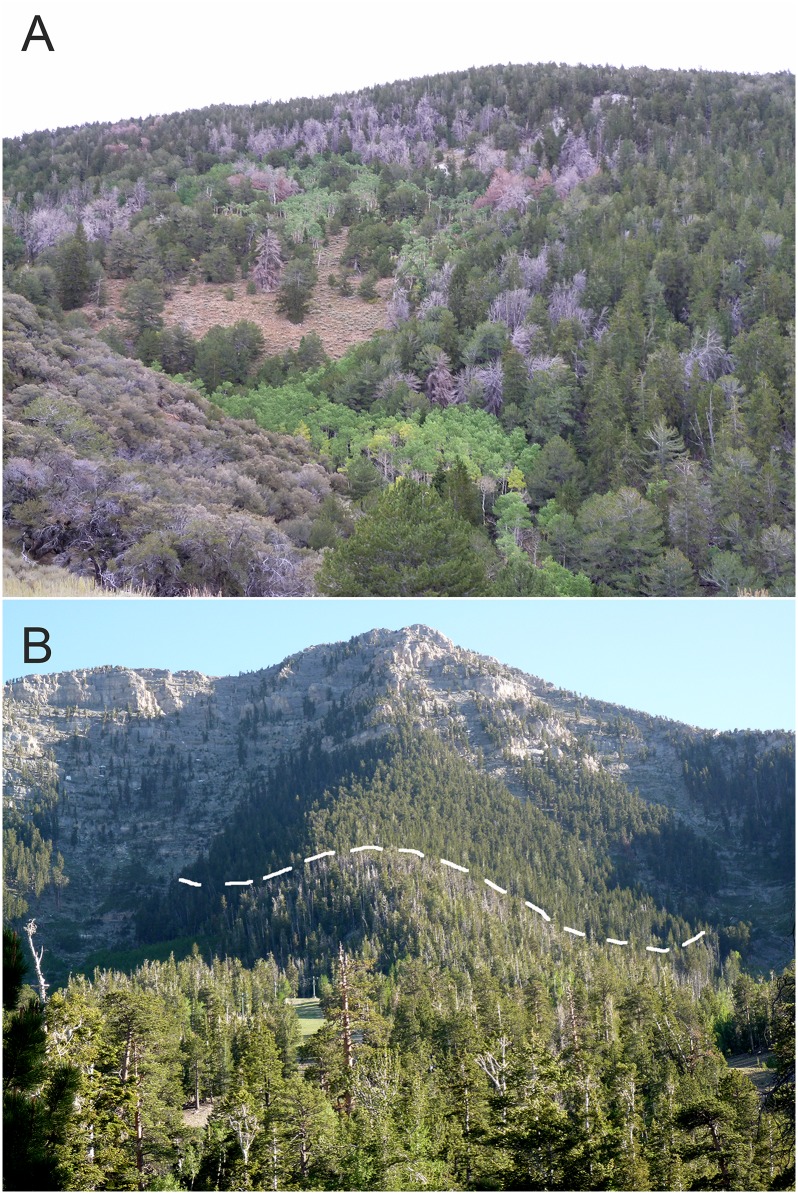
Photographs of limber pine (*Pinus flexilis*) and Great Basin bristlecone pine (*Pinus longaeva*) forests (a) on Cave Mountain in east-central Nevada, and (b) in the Spring Mountains in southern Nevada. These tree species co-occur in nearly equal abundance on and near the top of Cave Mountain (a), the dead trees (gray trees) are mountain pine beetle-killed limber pine. In the Spring Mountains (b), these tree species co-occur below dashed line, note many dead limber pine trees (gray trees), but a nearly pure stand of un-attacked bristlecone pine occurs above the dashed line.

In this study, we investigated whether pioneering female MPBs use VOCs to choose between the host limber pine (*Pinus flexilis* James) and the non-host Great Basin bristlecone pine (*Pinus longaeva* Bailey). Limber pine is a favored and highly-productive host of MPBs [21] and limber pine forests have experienced dramatic MPB mortality since the 1990s [1]. In contrast, Great Basin bristlecone pine has not been shown to be an acceptable host for MPB [6] and concrete records of successful MPB attack are lacking. These two species often occur together as the only tree species growing at or near alpine treeline in the Great Basin and Intermountain West of the USA (the “*P*. *flexilis*/*P*. *longaeva* Series” [22]). These high elevation pine forests provide important ecosystem services [23], including stabilizing soil, improving snow retention, pioneering regeneration of alpine sites after fire, habitat for wildlife, and facilitating growth of other tree species [24]. This study was spurred by our observations at several sites in Nevada where these species co-occur that many limber pines were killed by MPB whereas neighboring bristlecone pines were unattacked (Fig 1).

The objectives of this work were to 1) collect and analyze VOCs of co-occurring limber and bristlecone pines as potential foraging cues for the MPB, and 2) assess the behavioral responses of female MPBs to limber and bristlecone pine VOCs in a Y-tube olfactometer. We also explored the role of candidate individual volatile compounds in the behavioral response of MPBs. We hypothesized that VOCs differ between tree species and serve as a readily available cue that foraging MPBs can use in host finding.

## Materials and Methods

### Source and handling of insects and plants

Adult mountain pine beetles were obtained from two locations infested with MPB (separated by about 90 km) by felling two mature lodgepole pines (*Pinus contorta* Dougl.) infested with MPB larvae from the Bear River Range of Northern Utah (41.9705°, -111.5406°, elevation 2200 m) and from the Caribou-Targhee National Forest in Southern Idaho (42.7772°, -111.2735°, elevation 2040 m). Sections from the bole of the trees (~60 cm long) were transported to the US Forest Service’s Rocky Mountain Research Station laboratory in Logan, UT and ends sealed with paraffin wax to reduce desiccation. The sections were placed in Percival incubator cabinets (12 hours of light per 24-hour cycle) at room temperature (ca. 21°C) to facilitate larval development to the adult stage (approximately 70 to 80 days). Emerging adults were collected daily and placed in petri dishes with moistened filter paper and stored in a refrigerator at approximately 3°C until use. Gender was determined using characters on the seventh abdominal tergite [25]. Virgin females aged 5–15 days were randomly selected for Y-tube trials.

Foliage of limber pine and Great Basin bristlecone pine used in bioassays was collected from Notch Peak, UT (39.1486°, -113.4060°, elevation 2788 m) and Wheeler Peak, NV (38.9991°, -114.2990°, elevation 3181 m) by cutting branches approximately 50 cm in length from a randomly selected bristlecone pine and limber pine from each site, and refrigerating them in sealed plastic bags at approximately 3°C until use. VOCs from these samples were collected and analyzed at time of use as described below. The USDA Forest Service, Humboldt-Toiyabe and Caribou-Targhee National Forests, and the Utah Division of Forestry, Fire, and State Lands granted permission for use of all field sites.

### Collection and analysis of VOCs

VOCs were collected from co-occurring limber pine and bristlecone pine trees of similar size in the Spring Mountains near Las Vegas, Nevada (June 2013; 36.2935°, -115.6861°, elevation 2910 m) and on Cave Mountain near Ely, Nevada (August 2013; 39.1623°, -114.6109, elevation 3220 m). Trees of similar size were selected for sampling and VOCs were collected from lower branches (≤ 3 m above ground) which correspond to the heights at which dispersing MPBs fly [9]. The mean height of limber pine trees sampled was 12.1 ± 0.7 m and mean diameter at breast height (dbh) was 102 ± 11.6 cm. The mean height of bristlecone pine trees sampled was 12.9 ± 0.61 m and mean dbh was 85 ± 7.9 cm). Field collection of VOC emissions followed procedures described in Page et al. [26,27]. Approximately 70 cm of the apical portion of each branch on each tree was enclosed in a clear Teflon bag (50 cm wide x 75 cm deep; American Durafilm Co., Holliston, MA) and air was pulled (0.5 L/min) through VOC traps that contained 30 mg of the absorbent material HayeSep-Q (Restek, Bellefonte, Pennsylvania) using an automated portable VOC collection system (Volatile Assay Systems, Rensselaer, NY). VOCs were collected for 30 minutes after which the foliage from the enclosed portion of the branch was weighed to obtain a fresh weight.

VOCs were eluted from traps using 200 μl of dichloromethane and 1,000 ng of n-nonyl-acetate added as an internal standard. Samples were analyzed using an Agilent 7890A gas chromatograph (GC) coupled with a 5975C mass spectrometer and separated on a HP-1ms (30 m x 0.25 i.d, 0.25 μm film thickness) column; helium was used as the carrier gas. The GC oven was maintained at 35°C for 3 minutes and then increased by 5°C per minute to 125°C, then 25°C per minute to 250°C. Quantifications were made relative to internal standards using ChemStation software (Agilent Technologies, Wilmington, DE), and identifications of compounds confirmed by comparing retention times and mass spectra to commercial standards. Measurements of VOC emissions (ng per hour per gram) were on a fresh weight basis.

Statistical analyses for VOCs were performed using the non-parametric Kruskal-Wallis one-way analysis of variance [28] using R statistical software [29] to identify compounds with significant (P ≤ 0.05) differences, and to test whether samples originate from the same distribution.

### Y-tube olfactometer trials

We used a Y-tube olfactometer to investigate the response of adult female MPBs to airborne cues, following the methodology of others (e.g., [30], [31], [32]). Y-tube olfactometers have been widely used to examine the role of volatile cues in host location by flying arthropods, including bark beetles [32]. The Y-tube system (Sigma Scientific LLC, Micanopy, FL, USA) consisted of a 2-port Clean Air Delivery System (CADS-2P), inline odor source chambers (custom made), and a glass Y-tube (YT-2425). The CADS-2P provided flow-controlled, purified air via 0.64 cm outer diameter (OD) Teflon tubing to the odor source chambers (one chamber upwind of each Y-tube arm) and then the Y-tube. The glass odor source chambers were 19 cm long with 5.5 cm inner diameter (ID); the upstream end was sealed with a removable 5.5 cm OD Teflon o-ring endcap with 0.64 cm OD tubing connector, and the downstream end tapered to accept 0.64 cm OD Teflon tubing. The glass Y-tube had a 2.4 cm ID with 24/25 inner ground-glass joints on all ends, a 16 cm lower arm, and 10 cm upper arms that branch at an inner angle of approximately 75°. A specimen adapter (SA-2425), attached via ground-glass joint to the bottom of the Y-tube was used to introduce beetles to the airstream.

Trials were conducted in a greenhouse at temperatures between 20–27°C. Mountain pine beetles are positively phototactic [13], so to assure balanced lighting we placed the Y-tube in an open-top box that was lined with black felt (55 tall x 55 wide x 90 cm long). A greenhouse light (400W metal halide, Sylvania Inc., Manchester, NH, USA) was centered 1 m above and just beyond the apex of the Y-tube. To facilitate beetle walking, we placed a 16 cm long, 2 mm diameter metal wire in the bottom of the Y-tube, extending from the introduction point to the junction of the ‘Y’. The odor sources, 20 g of plant material (10–15 cm branches with attached needles) and/or rubber septa treated with VOCs, were placed in odor source chambers and an individual insect introduced via specimen adapter at the bottom of the Y-tube. Airflow was set at 0.5 L/min for all trials. Trials ended when the insect moved past the midpoint of the bifurcation in the Y-tube and 5 cm into one of the arms of the ‘Y’ or after 10 minutes if the insect did not respond (“no responses”). Individual beetles were only used once. The odor source chambers were alternated every five trials. For each odor source, trials were run until at least 25 choices were made (i.e. excluding no responses).

We used rubber septa treated with synthetic VOCs to test how individual compounds affect beetle behavior following methods outlined by Runyon et al. [33]. We chose to examine 3-carene and D-limonene because the relative amounts of these compounds differed greatly between bristlecone and limber pine, and they were commercially available in nearly pure form. Red rubber septa (6.6 mm O.D., Sigma Aldrich, St. Louis, MO, USA) were treated with 1 μg of either 3-carene (Product No: 21986, ≥98.5% sum of enantiomers, Sigma Aldrich) or D-limonene (Product No: 62118, ≥99% sum of enantiomers, Sigma Aldrich) in *n*-hexane (Macron Chemicals, Center Valley, PA, USA); 500 μl of 200 ng/μl hexane solution added to each septum. Control septa were treated with 500 μl of *n*-hexane only. Treated and control septa were left in a fume hood at room temperature and release rates checked each day as described above. Release rates of both compounds responded similarly: amounts released mimicked that of limber pine foliage used in Y-tube trials on day 4 after treatment for D-limonene (approximately 80 ng per hr) and day 5 after treatment for 3-carene (approximately 50 ng per hr) after treatment. We collected and analyzed a small number of foliage samples with the commercial compound added to verify that the target compound was present and in greater abundance.

Statistical analyses for Y-tube trials were performed using chi-square tests with the Yate’s continuity correction for small sample sizes [34]: for each trial we subtracted 0.5 from observed values greater than the expected and added 0.5 to observed values less than the expected.

## Results

### Great Basin bristlecone pine and limber pine VOCs

**Fig 2:**
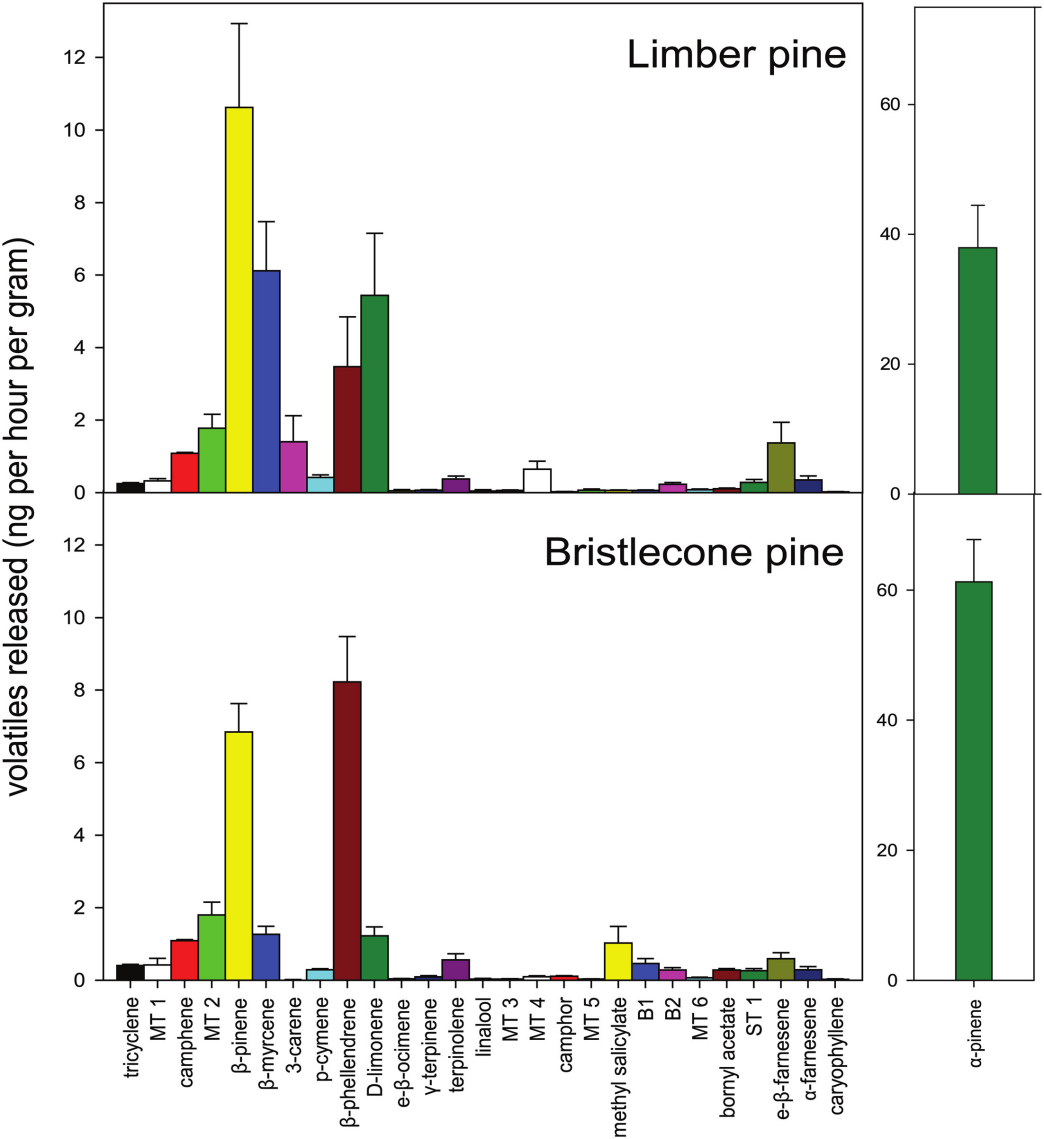
Total volatiles emitted (ng per hour per gram, *n* = 15) by limber pine (*Pinus flexilis*) and Great Basin bristlecone pine (*Pinus longaeva*) at Cave Mountain, Nevada. These tree species co-occur in nearly equal abundance at this site and many limber pines have been killed by mountain pine beetles whereas bristlecone pines have not been attacked. Note different scale for α-pinene.

**Fig 3:**
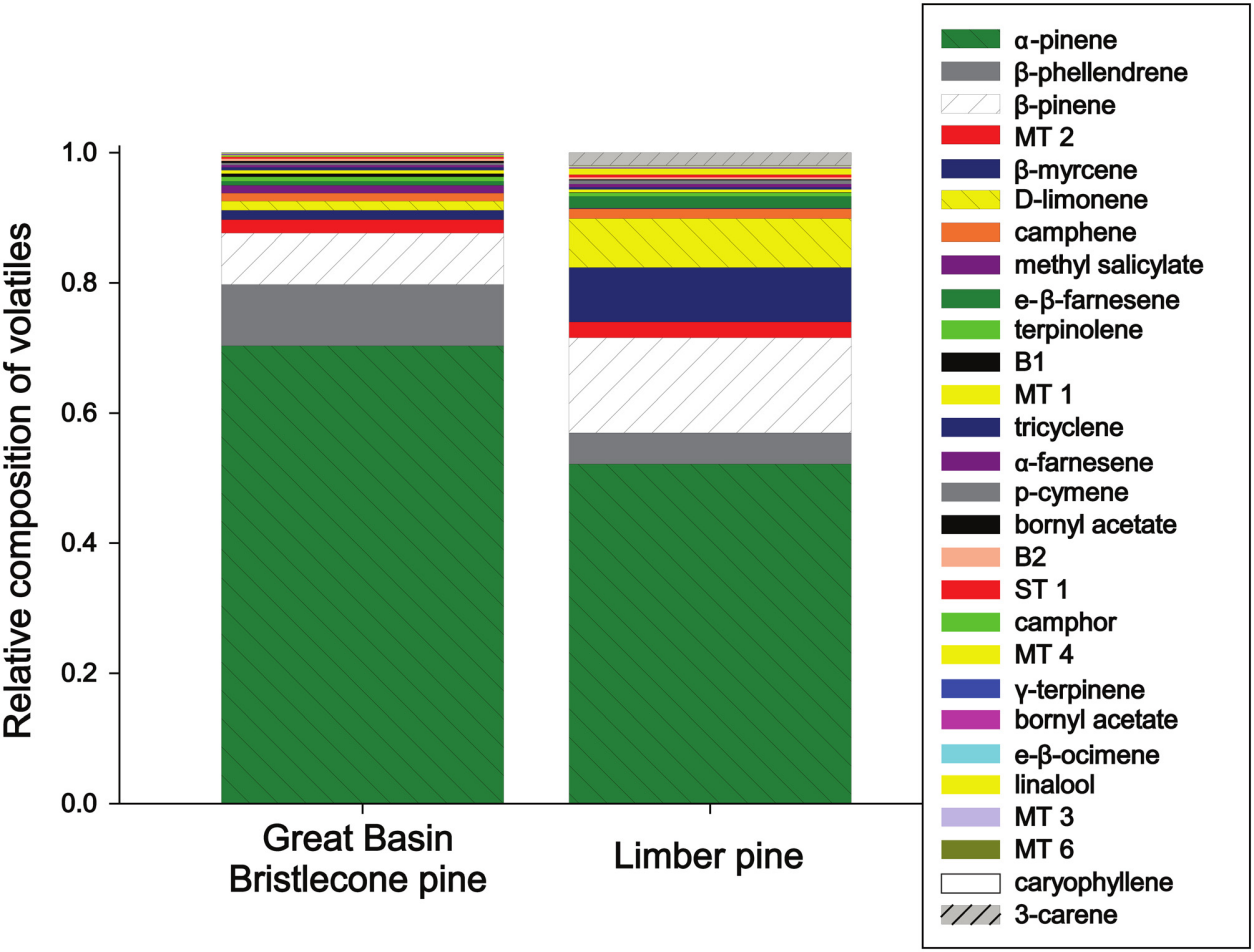
Relative composition of volatiles emitted by co-occurring limber pine (*Pinus flexilis*) and Great Basin bristlecone pine (*Pinus longaeva*) at Cave Mountain, Nevada. Compounds in the legend are listed from most abundant (top) to least abundant (bottom) emitted by Great Basin Bristlecone pine.

The VOCs emitted by Great Basin bristlecone pine and limber pine at Cave Mountain were similar. Both species emitted the same 28 VOCs (Fig 2, Fig 3 and Table 1) and differed in amounts produced for only ten of these compounds (*P* < 0.05; Table 1). Moreover, the total amount of VOCs released per gram of foliage did not differ between species (Table 1). Monoterpenes dominated the VOC composition of both tree species with α-pinene being the most abundant followed by β-pinene, β-phellandrene, D-limonene, and β-myrcene (Fig 2, Fig 3 and Table 1). A notable difference was the monoterpene 3-carene which was produced by limber (1.4 ± 0.72 ng per hour per gram) but nearly absent from bristlecone VOCs (0.02 ± 0.003 ng per hour per gram). The ratios of compounds also varied between species, for example the ratio of β-phellandrene to D-limonene was approximately 1:1 in limber but 7:1 in bristlecone (Fig 3, Table 1). The amounts and identity of VOCs reported here for Cave Mountain are very similar for co-occurring bristlecone and limber pine trees at a second site in the Spring Mountains near Las Vegas, Nevada (data not shown). We verified that VOCs from foliage used in Y-tube trials were similar to that of intact trees: clipped foliage emitted the same major compounds in approximately the same proportions, only in greater amounts per gram (perhaps due to clipping the branches off trees) (S1 Fig).

**Table 1:** Volatiles emitted (ng per hour per gram; *n* = 15) by co-occurring Great Basin bristlecone pine (*Pinus longaeva*) and limber pine (*Pinus flexilis*) at Cave Mountain, Nevada. Significant differences are highlighted in bold.

	Great Basin bristlecone		Limber		
Compound	Mean	SE	Mean	SE	P-value
**tricyclene**	**0.41**	**0.029**	**0.25**	**0.027**	**0.001**
MT 1	0.42	0.181	0.32	0.062	0.290
**α-pinene**	**61.25**	**6.473**	**37.95**	**6.533**	**0.011**
camphene	1.09	0.029	1.08	0.028	0.349
MT 2	1.79	0.357	1.77	0.386	0.604
β-pinene	6.85	0.784	10.62	2.316	0.481
**β-myrcene**	**1.26**	**0.221**	**6.12**	**1.357**	**<0.001**
**3-carene**	**0.02**	**0.003**	**1.40**	**0.721**	**<0.001**
p-cymene	0.29	0.034	0.42	0.072	0.254
**β-phellandrene**	**8.22**	**1.254**	**3.47**	**1.373**	**0.001**
D-limonene	1.22	0.245	5.44	1.716	0.120
e-β-ocimene	0.04	0.009	0.05	0.028	0.188
γ-terpinene	0.09	0.031	0.07	0.016	0.573
terpinolene	0.56	0.170	0.37	0.083	0.533
linalool	0.03	0.017	0.04	0.037	0.318
MT 3	0.03	0.007	0.06	0.019	0.382
**MT 4**	**0.10**	**0.025**	**0.65**	**0.225**	**0.001**
**camphor**	**0.11**	**0.014**	**0.03**	**0.005**	**<0.001**
MT 5	0.03	0.005	0.07	0.029	0.208
**methyl salicylate**	**1.02**	**0.459**	**0.06**	**0.015**	**<0.001**
**B1**	**0.46**	**0.137**	**0.06**	**0.016**	**<0.001**
B2	0.28	0.067	0.23	0.047	0.633
MT 6	0.07	0.014	0.08	0.018	0.983
**bornyl acetate**	**0.28**	**0.037**	**0.11**	**0.021**	**<0.001**
ST 1	0.26	0.060	0.29	0.080	0.647
e-β-farnesene	0.60	0.165	1.37	0.570	0.254
α-farnesene	0.29	0.091	0.35	0.111	0.480
caryophyllene	0.03	0.009	0.02	0.009	0.509
Total volatiles	87.11	7.891	72.76	12.400	0.110

MT = unidentified monoterpene, B = unidentified benzenoid, ST = unidentified sesquiterpene.

### Behavioral responses of female MPBs to VOCs

**Fig 4:**
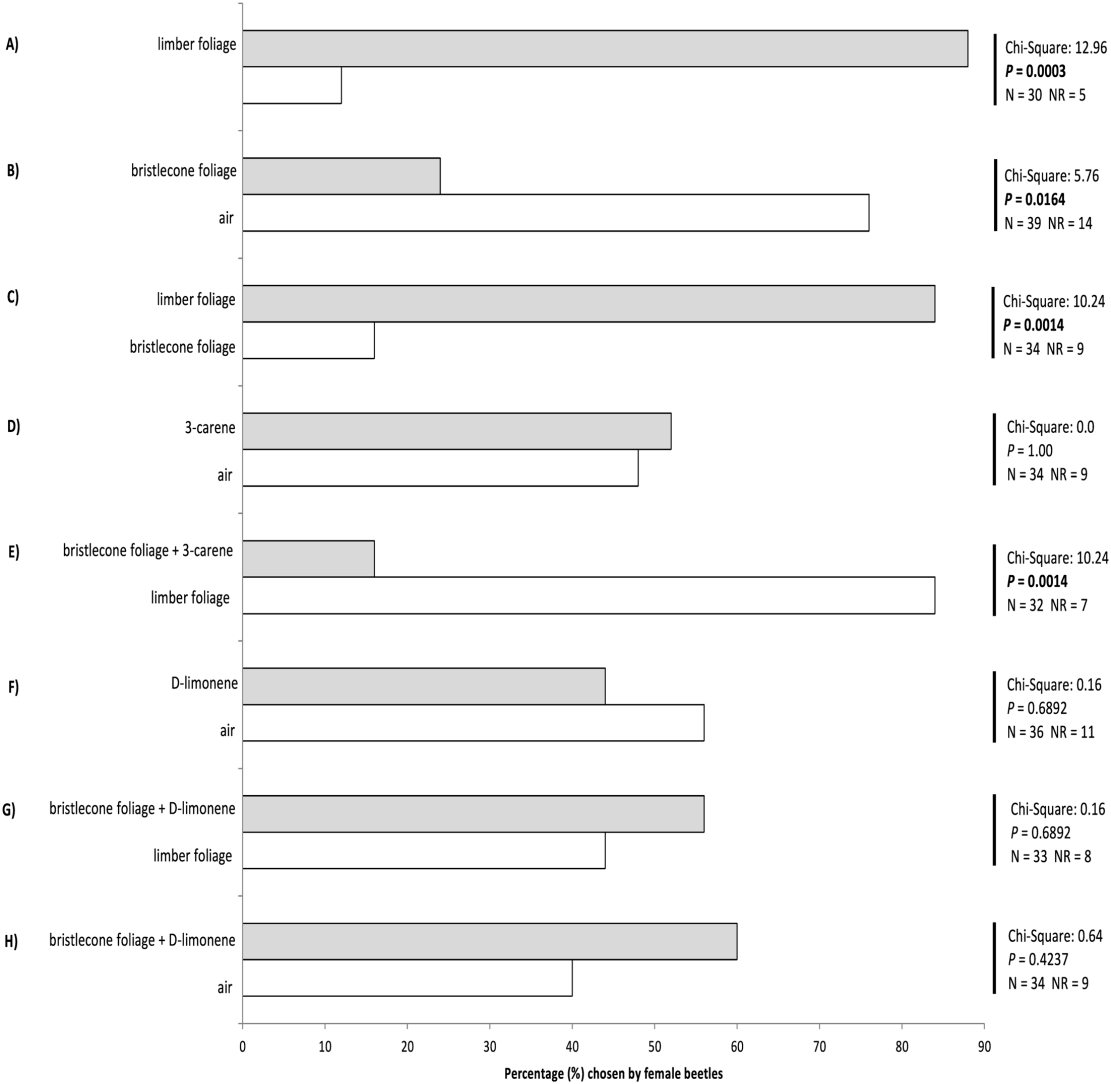
Behavioral responses of walking female mountain pine beetles (*Dendroctonus ponderosae*) to different odor sources in a Y-tube olfactometer. We used chi-square statistical tests for comparison between the numbers for each choice. NR = no response after 10 minutes. Significant results shown in bold. N = total number of trials (including no responses).

Adult female MPBs overwhelmingly chose the Y-tube arm with limber pine VOCs over the arm with clean air (22 limber vs. 3 air, 5 no responses; Fig 4A). In contrast, MPB females avoided bristlecone VOCs in favor of clean air (6 bristlecone vs. 19 air, 14 no responses; Fig 4B). When presented with VOCs from both limber and bristlecone, female MPBs overwhelmingly chose limber VOCs (21 limber vs. 4 bristlecone, 9 no responses; Fig 4C). We tested a role for 3-carene and D-limonene in the behavioral response by presenting the synthetic VOCs on rubber septa in the Y-tube. 3-carene had no effect on beetle behavior when presented alone (13 3-carene vs. 12 air, 7 no responses; Fig 4D) or when added to bristlecone pine VOCs (21 limber vs. 4 bristlecone + 3-carene, 7 no responses; Fig 4E). Similarly, D-limonene alone did not affect MPB behavior (11 D-limonene vs. 14 air, 11 no responses; Fig 4F). However, addition of D-limonene to bristlecone VOCs negated MPBs strong preference for limber VOCs (11 limber vs. 14 bristlecone + D-limonene, 9 no responses; Fig 4G) and blocked the ability of MPBs to avoid bristlecone VOCs (15 bristlecone + D-limonene, 10 air, 9 no responses; Fig 4H).

## Discussion

There is some debate about how pioneering female MPBs locate hosts with the dominant hypothesis being a combination of random landings and visual cues followed by direct assessment of host suitability after landing [8,35]. The explanation that pioneering females locate trees randomly [16,36], and/or using sight [13] gained support in part because it elegantly explains why large trees are disproportionately attacked—because they present beetles with the largest landing and visual targets. However, there is evidence in the literature that dispersing female MPBs use VOCs when foraging for hosts. Female MPBs were attracted to cages containing host material but not empty cages in the absence of normal visual cues [37] and antennae of female MPBs are capable of perceiving and responding to host tree VOCs [38]. Moreover, other bark beetle species are known to use VOCs to locate hosts, including other *Dendroctonus* species [20,34]. Here, the results from our study show that female MPBs are strongly attracted to VOCs emitted from limber pine, a preferred host, and are repelled by VOCs emitted from Great Basin bristlecone pine, a non-host. Moreover, female MPBs can distinguish limber and bristlecone pine trees using VOCs and preferentially move toward the former. These findings provide strong evidence that female MPBs use VOCs as cues to locate and select among potential hosts.

The VOCs of limber and bristlecone pine are very similar (Fig 2), so how do MPBs distinguish between them? We chose two candidate VOCs that differed between species and examined whether they are involved in host choice: the monoterpenes 3-carene and D-limonene. 3-carene alone or addition of 3-carene to bristlecone VOCs to mimic the amount in limber VOCs had no effect on beetle behavior (Fig 4D and 4E). However, similar addition of D-limonene to bristlecone VOCs blocked MPB’s ability to distinguish between trees species (Fig 4G). Interestingly, D-limonene alone was not attractive to MPBs (Fig 4F) suggesting that it is likely the combination or ratio of compounds that provides species-specific information to MPBs. The relative proportion of volatile components in a VOC blend is known to be used in host recognition by some insect herbivores [39], and experimentally enhancing levels of certain volatile components has been shown to interfere with host location of other herbivore species [40]. In fact, VOCs of many conifer species lack species-specific compounds, suggesting that bark beetles in general might detect differences in the ratios of different compounds to discriminate among tree species [41]. Recent research suggests that *Dendroctonus valens* LeConte, a species related to MPB, use small variations in ratios of VOCs to gauge and select large diameter trees over small diameter trees [32]. This provides a plausible mechanism by which beetles could measure and choose large host trees using VOCs alone, however, we expect that VOCs and visual cues both contribute to host location and selection by MPBs, as suggested by others [42,43].

The tree species examined in this study, Great Basin bristlecone pine and limber pine, are climax species that often co-occur as the only trees at or near alpine treeline across much of the Great Basin of North America [22]. Such high elevation ecosystems are of great ecological importance, but are rapidly declining across western North America due to unprecedented outbreaks of MPBs, climate change, and the non-native white pine blister rust [6,44]. Great Basin bristlecone pine is of particular interest because it is one of the longest-lived organisms on Earth, reaching ages approaching 5,000 years, and one of the most highly fragmented high elevation conifer species [45]. A better understanding of how MPBs locate and select hosts in high elevation systems will help us predict impacts and could allow development of tactics to manage MPBs in these important, at-risk communities. Moreover, the discovery that MPBs avoid bristlecone VOCs helps shed light on the great longevity of bristlecone pines. It is likely that bristlecone pines possess additional defense mechanisms to MPBs (e.g. phloem defensive chemistry) and that the VOCs provide long-distance cues about host quality to beetles.

In conclusion, we show that female MPBs use VOCs in the initial location and selection between limber and bristlecone pines and that D-limonene plays a role in concert with other unidentified compounds. Such a role for VOCs in host location by MPBs is not surprising given VOCs represent a readily-available cue for foraging beetles, and that MPBs utilize a sophisticated VOC communication system to coordinate mass attacks once hosts are located [12]. These findings beg more questions. We examined foliar VOCs since they should represent the largest odor source, but what about VOCs emitted from boles (the portion of a tree attacked by beetles), do they differ from foliar VOCs and are they used by MPBs? Which compounds and ratio of compounds are used by MPBs to find limber pines and avoid bristlecone pines? Are there common similarities and differences between VOCs of hosts and non-hosts that MPBs could use as general rules when searching for hosts? How do VOCs change with host condition and does this affect beetle’s choices? Finally, VOCs underlying mass attacks have been successfully used to manage MPBs [8] and the results presented here suggest that VOCs used in host location have been overlooked but might similarly be exploited for management of MPBs.

## Supporting Information

S1 Appendix VOC data for bristlecone pine versus limber pine trees at Cave Mountain, Nevada. VOCs reported in ng per hr per gram.
(XLSX)

S2 Appendix VOC data for bristlecone pine and limber pine foliage used in y-tube experiment. VOCs reported in ng per hr per gram.
(XLSX)

S1 Fig Total volatiles emitted (ng per hour per gram *n* = 4) by limber pine (*Pinus flexilis*) and Great Basin bristlecone pine (*Pinus longaeva*) clipped foliage used in Y-tube trials. Note different scale for α-pinene.
(EPS)

## References

[1] Man G. Major forest insect and disease conditions in the United States [Internet]. 2010. Available: http://www.fs.fed.us/foresthealth/publications/ConditionsReport_2009.pdf.

[2] Saab VA, Latif QS, Rowland MM, Johnson TN, Chalfoun AD, Buskirk SW, et al Ecological consequences of mountain pine beetle outbreaks for wildlife in western North American forests. For Sci. 2014;60: 539–559.

[3] Hansen EM. Forest development and carbon dynamics after mountain pine beetle outbreaks. For Sci. 2014;60: 476–488.

[4] Griffin JM, Turner MG, Simard M. Nitrogen cycling following mountain pine beetle disturbance in lodgepole pine forests of Greater Yellowstone. For Ecol Manage. 2011;261: 1077–1089. 10.1016/j.foreco.2010.12.031

[5] Jenkins MJ, Runyon JB, Fettig CJ, Page WG, Bentz BJ. Interactions among the mountain pine beetle, fires, and fuels. For Sci. 2014;60: 489–501. 10.5849/forsci.13-017

[6] Gibson KE, Skov K, Kegley S, Jorgensen C, Smith S, Witcosky J. Mountain pine beetle impacts in high-elevation five-needle pines: current trends and challenges. US Department of Agriculture, Forest Service, Forest Health Protection Washington, DC, USA; 2008.

[7] Logan JA, Macfarlane WW, Willcox L. Whitebark pine vulnerability to climate-driven mountain pine beetle disturbance in the Greater Yellowstone Ecosystem. Ecol Appl. 2010;20: 895–902. 10.1890/09-0655.1 20597278

[8] Progar RA, Gillette N, Fettig CJ, Hrinkevich K. Applied chemical ecology of the mountain pine beetle. For Sci. 2014;60: 414–433.

[9] Pitman GB, Vité JP, Kinzer GW. Bark beetle attractants: trans-verbenol isolated from *Dendroctonus* Nature. Nature Publishing Group; 1968 10.1038/218168a0

[10] Borden JH, Pureswaran DS, Lafontaine JP. Synergistic blends of monoterpenes for aggregation pheromones of the mountain pine beetle (Coleoptera: Curculionidae) J Econ Entomol. The Oxford University Press; 2008;101: 1266–1275. 10.1093/jee/101.4.1266 18767736

[11] Hunt DWA, Borden JH, Lindgren BS, Gries G. The role of autoxidation of α-pinene in the production of pheromones of *Dendroctonus ponderosae* (Coleoptera: Scolytidae) Can J For Res. NRC Research Press; 1989;19: 1275–1282. 10.1139/x89-194

[12] Safranyik L, Carroll AL. The biology and epidemiology of the mountain pine beetle in lodgepole pine forests The mountain pine beetle: a synthesis of biology, management and impacts on lodgepole pine. Canadian Forest Service; 2007; 3–66.

[13] Shepherd RF. Factors influencing the orientation and rates of activity of *Dendroctonus ponderosae* Hopkins (Coleoptera: Scolytidae). Can Entomol. 1966;98: 507–518. 10.4039/Ent98507-5

[14] Billings RF, Gara RI, Hrutfiord BF. Influence of ponderosa pine resin volatiles on the response of *Dendroctonus ponderosae* to synthetic trans-verbenol. Environ Entomol. 1976;5: 171–179. 10.1093/ee/5.1.171

[15] Burnell D. A dispersal-aggregation model for mountain pine beetle in lodgepole pine stands. Res Popul Ecol. 1977; 19: 99–106. 10.1007/BF02510942

[16] Hynum BG, Berryman AA. *Dendroctonus ponderosae* (Coleoptera: Scolytidae): pre-aggregation landing and gallery initiation on lodgepole pine. Can Entomol. 1980;112: 185–191. 10.4039/Ent112185-2

[17] Wood D. The role of pheromones, kairomones, and allomones in the host selection and colonization behavior of bark beetles. Annu Rev Entomol. 1982 10.1146/annurev.en.27.010182.002211

[18] Borden J, Hunt D, Miller DR, Slessor KN. Orientation in forest Coleoptera: an uncertain outcome of responses by individual beetles to variable stimuli Payne T, Birch M, Kennedy C, editors. Mechanisms in insect olfaction. 1986.

[19] Raffa K, Phillips T, Salom S. Strategies and mechanisms of host colonization by bark beetles. Beetle-pathogen Interactions in conifer forests. 1993; 103–120.

[20] Rudinsky JA. Scolytid beetles associated with Douglas fir: Response to terpenes. Science 1966;152: 218–219. 10.1126/science.152.3719.218 17741636

[21] Langor DW. Host effects on the phenology, development, and mortality of field populations of the mountain pine beetle, *Dendroctonus ponderosae* Hopkins (Coleoptera: Scolytidae). Can Entomol. 1989;121: 149–157.

[22] Youngblood AP, Mauk RL. Coniferous forest habitat types of central and southern Utah. 1985; USDA Forest Service, Intermountain Research Station, General Technical Report INT-187, 89 pp.

[23] Schoettle AW. Ecological roles of five-needle pines in Colorado: potential consequences of their loss Breeding and genetic resources of five-needle pines: growth, adaptability, and pest resistance. Edited by Sniezko RA, Samman S, Schlarbaum SE, Kriebel HB USDA For Serv Proc RMRS-P-32. 2004; 124–135.

[24] Baumeister D, Callaway RM. Facilitation by Pinus flexilis during succession: a hierarchy of mechanisms benefits other plant species. Ecology. 2006; 87: 1816–1830. 10.1890/0012-9658(2006)87[1816:FBPFDS]2.0.CO;2 16922330

[25] Lyon RL. A useful secondary sex character in Dendroctonus bark beetles. Can Entomol. 1958;90: 582–584. 10.4039/Ent90582-10

[26] Page WG, Jenkins MJ, Runyon JB. Mountain pine beetle attack alters the chemistry and flammability of lodgepole pine foliage. Can J For Res. 2012;42: 1631–1647. 10.1139/x2012-094

[27] Page WG, Jenkins MJ, Runyon JB. Spruce beetle-induced changes to Engelmann spruce foliage flammability. For Sci. 2014;60: 691–702. 10.5849/forsci.13-050

[28] Kruskal WH, Wallis WA. Use of ranks in one-criterion variance analysis. J Am Stat Assoc. 1952;47: 583–621.

[29] R Devolopment Core Team. R: A language and environment for statistical computing. R Foundation for Statistical Computing. Vienna, Austria. ISBN 3-900051-07-0, Available: http://www.r-project.org; 2012.

[30] Daisy BH, Strobel GA, Castillo U, Ezra D, Sears J, Weaver DK, et al Naphthalene, an insect repellent, is produced by Muscodor vitigenus, a novel endophytic fungus. Microbiology. 2002;148: 3737–3741. 12427963 10.1099/00221287-148-11-3737

[31] Piesik D, Weaver DK, Runyon JB, Buteler M, Peck GE, Morrill WL. Behavioural responses of wheat stem sawflies to wheat volatiles. Agric For Entomol. 2008;10: 245–253. 10.1111/j.1461-9563.2008.00380.x

[32] Liu Z, Wang B, Xu B, Sun J. Monoterpene variation mediated attack preference evolution of the bark beetle *Dendroctonus valens* . PLoS One 2011;6: e22005 10.1371/journal.pone.0022005 21811555 PMC3139614

[33] Runyon JB, Mescher MC, De Moraes CM. Volatile chemical cues guide host location and host selection by parasitic plants. Science 2006; 313: 1964–1967. 10.1126/science.1131371 17008532

[34] Sokal RR, Rohlf FJ. Biometry: the principles and practice of statistics in biological research 2nd edition. WH Freeman 1981.

[35] Safranyik L, Shore TL, Carroll AL, Linton DA. Bark beetle (Coleoptera: Scolytidae) diversity in spaced and unmanaged mature lodgepole pine (Pinaceae) in southeastern British Columbia. For Ecol Manage. 2004;200: 23–38. 10.1016/j.foreco.2004.06.004

[36] Vité JP, Gara RI. Volatile attractants from ponderosa pine attacked by bark beetles (Coleoptera: Scolytidae). Contrib Boyce Thompson Inst. 1962;21: 3.

[37] Moeck HA, Simmons CS. Primary attraction of mountain pine beetle, *Dendroctonus ponderosae* Hopk. (Coleoptera: Scolytidae), to bolts of lodgepole pine. Can Entomol. 1991;123: 299–304.

[38] Pureswaran DS, Gries R, Borden JH. Antennal responses of four species of tree-killing bark beetles (Coleoptera: Scolytidae) to volatiles collected from beetles, and their host and nonhost conifers. Chemoecology 2004;14: 59–66. 10.1007/s00049-003-0261-1

[39] Bruce TJA, Pickett JA. Perception of plant volatile blends by herbivorous insects—finding the right mix. Phytochemistry 2011;72: 1605–1611. 10.1016/j.phytochem.2011.04.011 21596403

[40] Visser JH, Avé DA. General green leaf volatiles in the olfactory orientation of the Colorado beetle, *Leptinotarsa decemlineata* . Entomol Exp Appl. 1978;24: 738–749. 10.1111/j.1570-7458.1978.tb02838.x

[41] Pureswaran DS, Gries R, Borden JH. Quantitative variation in monoterpenes in four species of conifers. Biochem Syst Ecol. 2004;32: 1109–1136. 10.1016/j.bse.2004.04.006

[42] Campbell SA, Borden JH. Integration of visual and olfactory cues of hosts and non-hosts by three bark beetles (Coleoptera: Scolytidae). Ecol Entomol. 2006;31: 437–449. 10.1111/j.1365-2311.2006.00809.x

[43] Campbell SA, Borden JH. Additive and synergistic integration of multimodal cues of both hosts and non-hosts during host selection by woodboring insects. Oikos. 2009;118: 553–563. 10.1111/j.1600-0706.2009.16761.x

[44] Tomback DF, Achuff P. Blister rust and western forest biodiversity: ecology, values and outlook for white pines. For Pathol. 2010;40: 186–225. 10.1111/j.1439-0329.2010.00655.x

[45] Stritch L, Mahalovich M, Nelson KG. 2011. *Pinus longaeva* The IUCN Red List of Threatened Species. Version 2015.2. [Internet]. Available: http://www.iucnredlist.org/details/34024/0.

